# Exploring the Adsorption Features of Furan and 1,*n*‐Dioxane as Environmental Toxins on Two‐Dimensional RuC Nanosheet: A DFT Study

**DOI:** 10.1002/open.202400415

**Published:** 2025-01-29

**Authors:** Amna H. M. Mahmoud, Muhrail E. S. Aziz, Abdallah I. M. Rabee, Mohamed A. El‐Tayeb, Gamal A. H. Mekhemer, Tamer Shoeib, Mahmoud A. A. Ibrahim

**Affiliations:** ^1^ Computational Chemistry Laboratory Chemistry Department Faculty of Science Minia University Minia 61519 Egypt; ^2^ Leibniz-Institut für Katalyse Albert-Einstein-Str. 29 A 18059 Rostock Germany; ^3^ Department of Botany and Microbiology College of Science King Saud University P.O. Box 2455 Riyadh 11451 Saudi Arabia; ^4^ Department of Chemistry The American University in Cairo New Cairo 11835 Egypt; ^5^ School of Health Sciences University of KwaZulu-Natal Westville Campus Durban 4000 South Africa

**Keywords:** two-dimensional nanomaterials, environmental toxins, DFT calculations, adsorption features, RuC nanosheet

## Abstract

The potential of the two‐dimensional ruthenium carbide (RuC) nanosheet to detect highly toxic environmental compounds – namely, Furan (Fur) and 1,*n*‐Dioxane (1,*n*‐Diox) – was investigated utilizing the density functional theory (DFT) approach. The adsorption features of the Fur and 1,*n*‐Diox molecules on the RuC nanosheet were evaluated in parallel and vertical configurations. From energetic manifestations, Fur and 1,*n*‐Diox molecules preferred to be adsorbed in the parallel configuration rather than the vertical one on the RuC nanosheet with negative *E*_ads_ values of −27.80 and −9.30 kcal/mol, respectively, for Fur⋅⋅⋅RuC complexes. Bader charge findings demonstrated an electron‐accepting property for the Fur and 1,*n*‐Diox molecules during the adsorption process over the RuC nanosheet, as indicated by positive *Q*_t_ values. From the FMO findings, the *E*_HOMO_ and *E*_LUMO_ values of Fur/1,*n*‐Diox molecules, and the pure RuC nanosheet varied considerably after the adsorption process in both configurations. The band structure and TDOS/PDOS plots of Fur/1,*n*‐Diox⋅⋅⋅RuC complexes showed new bands and peaks for the RuC nanosheet after the adsorption process, proving the capability of the RuC nanosheet to detect the investigated small molecules. The outcomes of the current work can serve as a foundation for using the RuC nanosheets to detect highly toxic small molecules.

## Graphical abstract

This work investigates the potential of RuC nanosheet to detect highly toxic environmental compounds – namely, Furan (Fur) and 1,*n*‐Dioxan (1,*n*‐Diox) – using DFT methods. The energetic manifestations demonstrate the preferability of the parallel configuration in the adsorption of Fur/1,*n*‐Diox molecules over the RuC nanosheet than the vertical one. The Fur⋅⋅⋅RuC complex exhibits the highest negative adsorption energy value of −27.80 kcal/mol.

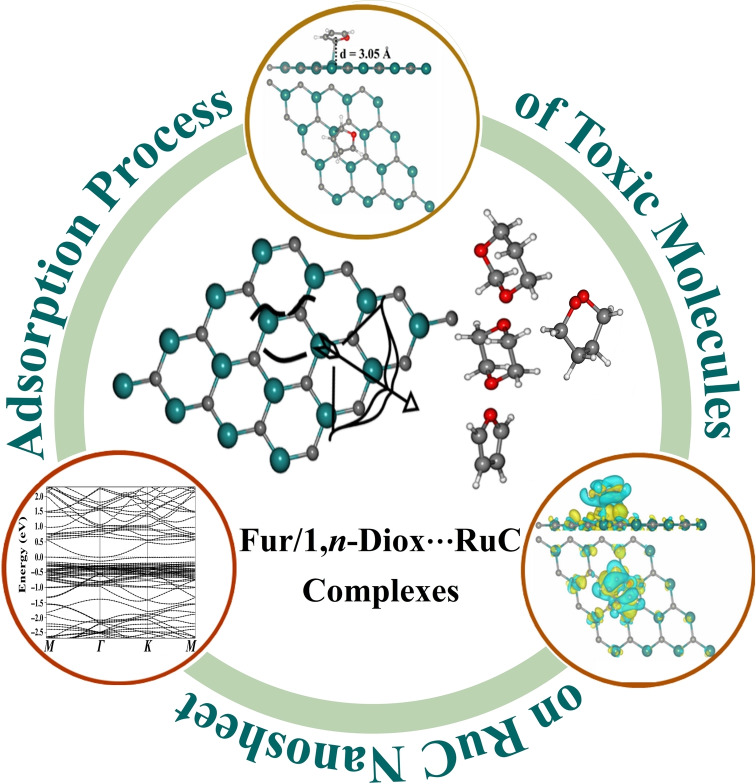

## Introduction

Two‐dimensional (2D) nanomaterials have drawn great interest due to their fundamental mechanical, electrical, and optical properties, making them industry‐wide prospective materials.[^1^, ^2^, ^3^, ^4^, ^5^, ^6^] Owing to the abovementioned characteristics, 2D nanomaterials have been employed as spintronic devices, gas sensors, and sources of energy.[^7^, ^8^, ^9^, ^10^] In this regard, numerous 2D nanomaterials have been synthesized as active materials, such as germanene,[^11^, ^12^] phosphorene,[^13^, ^14^] silicene,[^15^, ^16^] borophene,[^6^, ^17^, ^18^, ^19^] transition‐metal dichalcogenides (TMDs),[^20^, ^21^] and molybdenum disulfide.[^22^, ^23^, ^24^] Moreover, interest in developing 2D materials containing group III–IV elements and transition metals has grown significantly.[^25^, ^26^] Within this framework, the extraordinary properties of transition metal carbides (TMCs), such as superconductivity, catalysis, and energy storage, have attracted plenty of focus.^[27]^ Recently, a novel 2D TMC structure called ruthenium carbide (RuC) was predicted by applying density functional theory (DFT) calculations.^[28]^ The RuC nanosheet was found to create a hexagonal honeycomb structure with planar geometry that was highly retained at a temperature of 1000 K.^[28]^ Additionally, the RuC was shown to have exceptional electrical, structural, and mechanical stabilities.^[28]^ Due to its remarkable characteristics, the RuC nanosheet became a viable 2D material for sensing applications.^[29]^ For example, sensing of NO_2_, NH_3_, and NO molecules by RuC nanosheet was investigated using DFT methods.^[29]^ The findings revealed that the RuC nanosheet had a significant capacity to adsorb the NO molecule with adsorption energy (*E*_ads_) of −1.718 eV.^[29]^ However, the adsorption characteristics of RuC nanosheet, especially toward toxic molecules, have not been well investigated.

Environmental pollution is one of the most significant concerns influencing human health.^[30]^ Furan (C_4_H_4_O), a highly volatile and colorless five‐membered ring molecule, is typically found in the contaminating components of coal‐derived liquid.[^31^, ^32^] It is categorized as a potential carcinogen for humans based on the International Agency for Research on Cancer (IARC), which can be caused by the Maillard reaction.[^33^, ^34^, ^35^] Most known tumors caused by Furan are adenocarcinomas, accounting for about 90 % of cases; squamous cell carcinomas account for the remaining 10 %.^[36]^ Nonetheless, Furan is still used for the production of pyrrole derivatives, 2,2‐difuryl propane, a‐acetylfuran, and 2,2‐di(tetrahydrofurfuryl) propane.^[37]^ Furthermore, Furan is used as an industry‐wide metal surface corrosion inhibitor.^[38]^ 1,4‐Dioxane, a heterocyclic organic compound, has various industrial applications. Because of its ability to accept protons, 1,4‐Dioxane can be used as a solvent, a wetting agent in the textile process, a solvent stabilizer, and a chemical intermediate.^[39]^ According to the IARC, 1,4‐Dioxane is categorized as probably carcinogenic to humans (Group 2B carcinogens).^[40]^ Exposure to high doses of 1,4‐Dioxane can irritate the skin, eyes, and respiratory systems, as well as have harmful effects on the nervous system, liver, and kidneys.^[39]^ In the industrial sector, cement dust is considered one of the chemical toxins, and it contains a variety of metal oxides and organic compounds like 1,4‐Dioxane and Furan.^[41]^ Based on reports, employees in the cement industry exposed to dust for extended periods are more likely to get dyspnea than those who take preventative measures.[^42^, ^43^] As a result, eradicating and controlling Furan and 1,4‐Dioxane from contaminated sources have received increasing attention.[^44^, ^45^, ^46^, ^47^]

Therefore, for the first time, the present work investigated the adsorption of the Furan (Fur) and 1,*n*‐Dioxane (1,*n*‐Diox; *n*=2, 3, and 4) as environmental toxic compounds on the RuC nanosheet using several DFT calculations. In addition to executing the geometric structures, *E*_ads_ calculations improved the understanding of the interaction of the Fur and 1,*n*‐Diox molecules with the RuC nanosheet. Furthermore, the frontier molecular orbital (FMO), density of state (DOS), Bader charge transfer, and band structures calculations were executed. The current study may provide a significant opportunity to advance studying the potentiality of the RuC nanosheet as a sensing material for toxic compounds.

## Computational Methodology

All calculations were performed using DFT method implemented in the Quantum Espresso 7.1.[^48^, ^49^] The Perdew‐Burke‐Ernzerhof (PBE) method combined with the generalized gradient approximation (GGA) described the electron exchange‐correlation function.^[50]^ The electron‐core interaction was represented by the projector augmented wave (PAW) pseudo‐potential.^[51]^ The Grimme‐D3 method was used for accounting van der Waals corrections in all computations.[^52^, ^53^] The 50 and 400 Ry values were utilized for the energy and charge density cutoffs, respectively. The convergence thresholds of 10^−4^ eV/Å and 10^−5^ eV were used for the atomic force and total energy, respectively. As per the Monkhorst‐Pack mesh, the first Brillouin zone sampling within the geometry relaxation and density of state calculations was conducted using the 5×5 ×1 and 15×15×1 *k*‐points grids, respectively. Convergence was enhanced by applying the Marzari‐Vanderbilt smearing approach.^[54]^ To avoid interlayer contact along the *z*‐direction of the RuC nanosheet, a vacuum thickness of 20 Å was applied. A 4×4×1 supercell of the RuC nanosheet with 32 atoms was modeled to investigate the adsorption process of Fur and 1,*n*‐Diox (where *n*=2, 3, and 4) molecules. The *E*_ads_ of the Fur/1,*n*‐Diox⋅⋅⋅RuC complexes in vertical and parallel configurations were evaluated as follows:

(1) $$E_{ads\;}=E_{Fur/1,n-Diox\cdot \cdot \cdot RuC}-\left({E_{Fur/1,n-Diox}+E}_{RuC}\right)$$

where $E_{Fur/1,n-Diox\cdot \cdot \cdot RuC}$ , $E_{Fur/1,n-Diox}$ , and $E_{RuC}$ are the energies of total adsorption system, adsorbed molecules, and RuC nanosheet, respectively. The lowest unoccupied molecular orbitals (*E*_LUMO_) and highest occupied molecular orbitals (*E*_HOMO_) energies for the most stable Fur/1,*n*‐Diox⋅⋅⋅RuC complexes were determined within the FMO theory. The energy gap (*E*_gap_) values were estimated using the subsequent Equation:

(2) $$E_{gap}=E_{LUMO}-E_{HOMO}$$

The amount of charge transferred to or from the RuC nanosheet was evaluated using the Bader charge transfer method[^55^, ^56^] as follows:

(3) $$Q_{t}=Q_{combined\;RuC}-Q_{isolated\;RuC}$$

where the $Q_{combined\;RuC}$ and $Q_{isolated\;RuC}\;$ are the charges of the RuC nanosheet following and prior to the adsorption process, respectively. The charge density difference maps (Δ*ρ*) were constructed using this Equation:

(4) $$\Delta \rho =\rho_{Fur/1,n-Diox\cdot \cdot \cdot RuC}-\rho_{Fur/1,n-Diox}-\rho_{RuC}$$

where the $\rho_{Fur/1,n-Diox\cdot \cdot \cdot RuC}$ , $\rho_{Fur/1,n-Diox}$ , and $\rho_{RuC}$ are the charge densities of the total adsorption system, Fur/1,*n*‐Diox molecules, and RuC, respectively. The VESTA software was used to plot the charge density maps.^[57]^ In order to investigate the desorption process of Fur and 1,*n*‐Diox molecules from the RuC nanosheet, the recovery time (*τ*) for the investigated Fur/1,*n*‐Diox⋅⋅⋅RuC complexes was calculated as follows:
(5)

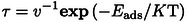




where the values of the Boltzmann constant (K), temperature (T), and attempt frequency (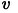 ) are 0.00199 kcal/mol.K, 298.15/ 310.15 K, and 10^12^ s^−1^, respectively. To understand the impact of the adsorption of Fur/1,*n*‐Diox molecules on the electronic characteristics of the RuC nanosheet, density of state (DOS) calculations were performed. The computational approach of the current work was developed and effectively applied in several previous studies.[^6^, ^18^, ^58^, ^59^]

## Results and Discussion

### Geometric Structures

**Figure 1:**
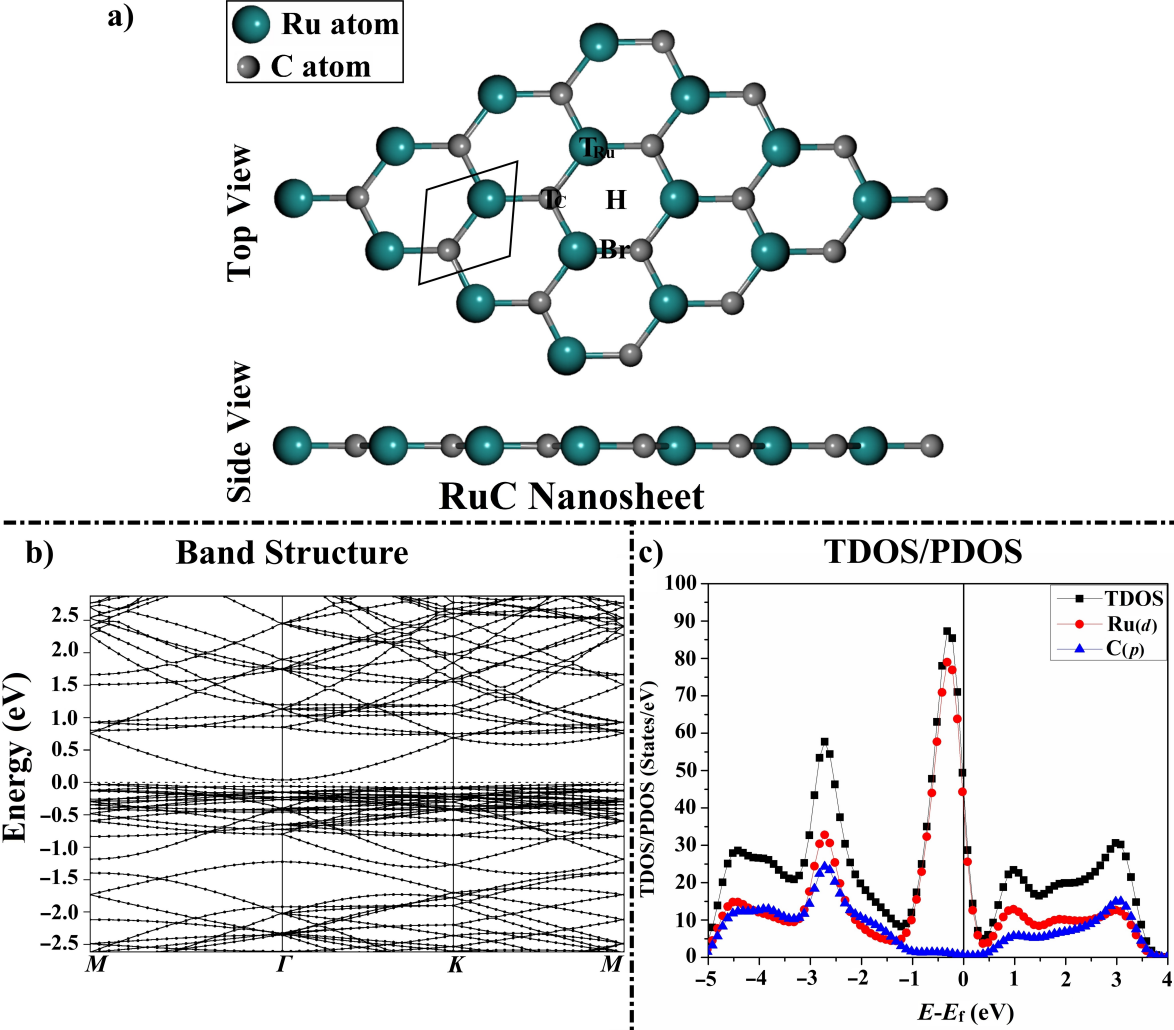
a) Top and side views of the 4×4×1 supercell of the RuC nanosheet. The black rhombus represents the RuC unit cell. The top, bridge, and hollow adsorption sites are denoted by the symbols T, Br, and H, respectively. b) Electronic band structure along the high symmetry path of the Brillouin zone (*M‐Γ‐K‐M*) and c) TDOS/PDOS for the pure RuC nanosheet. The Fermi level is set at zero energy.

After the relaxation of the RuC unit cell, the lattice constant was *a*=*b*=3.266 Å, which is consistent with the results of the earlier studies.[^28^, ^29^] Upon the relaxed unit cell, a 4×4×1 supercell of the RuC nanosheet was modeled and relaxed prior to the adsorption of the Fur and 1,*n*‐Diox molecules (Figure 1, Table S1). On the optimized structure of the RuC nanosheet, four adsorption sites were detected, represented as two top (T_Ru_ and T_C_) sites, the bridge (Br) site, and the hollow (H) site (Figure 1).

### Adsorption Energy

**Figure 2:**
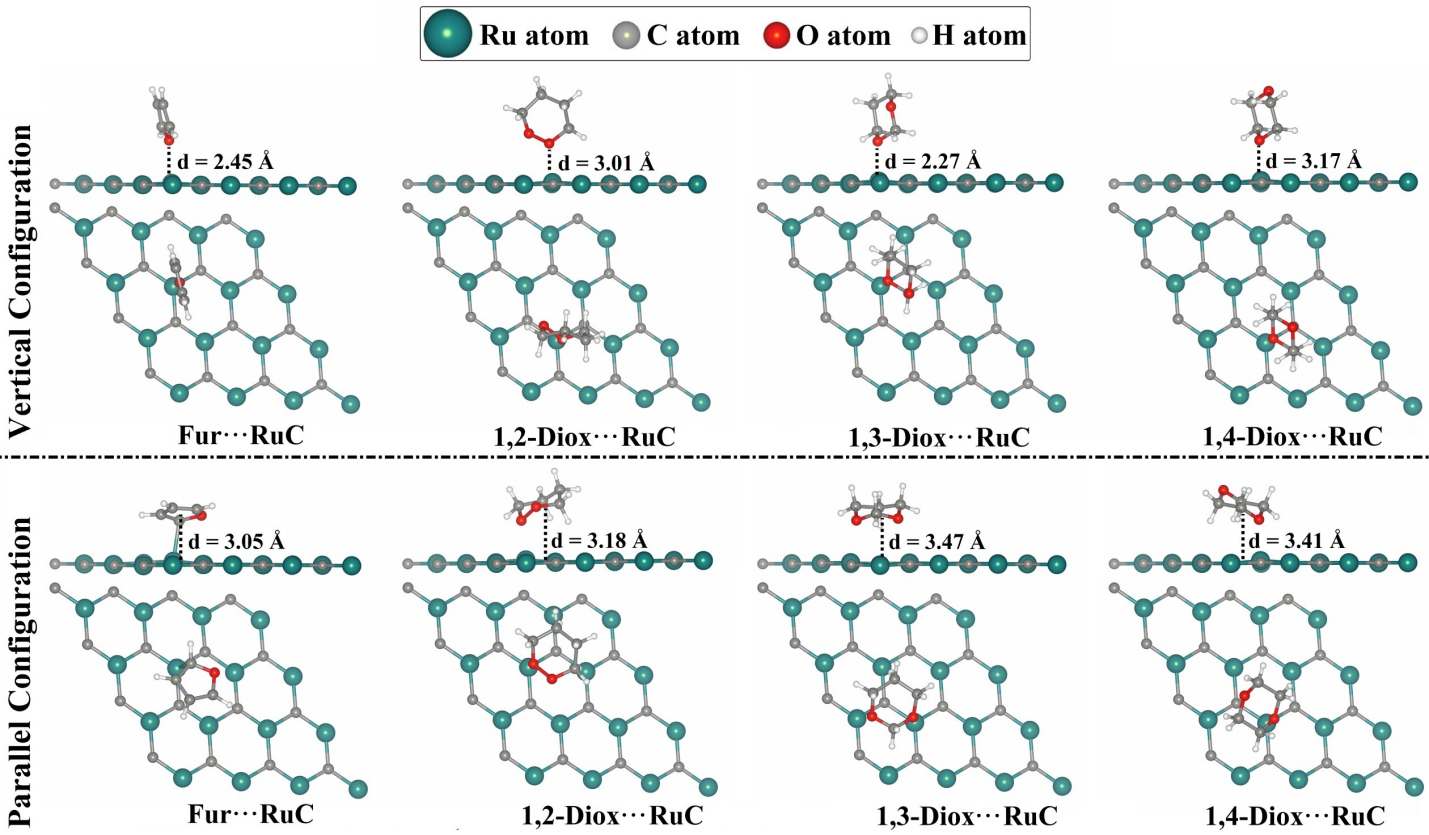
Side and top perspectives of the relaxed Fur/1,*n*‐Diox⋅⋅⋅RuC complexes (where *n*=2, 3, and 4) in vertical and parallel configurations. The equilibrium distances (d, Å) were measured between the RuC nanosheet and the oxygen atom facing its surface in the vertical configuration and between the RuC nanosheet and the center of the ring of the Fur/1,*n*‐Diox molecules in the parallel configuration.

The adsorption of Fur and 1,*n*‐Diox molecules was investigated in the parallel and vertical configurations with respect to the RuC nanosheet at all possible adsorption sites (Figure S1). The adsorption energies (*E*_ads_) and the equilibrium distances for all relaxed Fur/1,*n*‐Diox⋅⋅⋅RuC complexes were computed (Table S2). Structures of the most stable relaxed Fur/1,*n*‐Diox⋅⋅⋅RuC complexes and their atomic coordinates are represented in Figure 2 and Table S2, respectively. Based on the relaxed structures, the corresponding *E*_ads_ were calculated and are presented in Table 1. According to the results shown in Table 1, all Fur/1,*n*‐Diox⋅⋅⋅RuC complexes in both vertical and parallel configurations had negative *E*_ads_ values, verifying that the adsorption process occurred.

**Table 1:** Adsorption energies (*E*_ads_, kcal/mol) and equilibrium distances (d, Å) of the relaxed Fur/1,*n*‐Diox⋅⋅⋅RuC complexes (where *n*=2, 3, and 4) in the vertical and parallel configurations. Charge transfer difference (*Q*_t_, *e*) for the RuC nanosheet prior to and after the adsorption process.

System	Vertical Configuration			Parallel Configuration		
	*E*_ads_	d	*Q*_t_^[a]^	*E*_ads_	d	*Q*_t_^[a]^
Fur⋅⋅⋅RuC	−9.77	2.45	0.0187	−27.80	3.05	0.1581
1,2‐Diox⋅⋅⋅RuC	−16.27	3.01	0.0473	−20.53	3.18	0.0513
1,3‐Diox⋅⋅⋅RuC	−19.91	2.27	0.0766	−22.17	3.47	0.0776
1,4‐Diox⋅⋅⋅RuC	−19.85	3.17	0.0796	−20.88	3.41	0.0792

[a] *Q*_t_ was calculated based on Equation (3).

In the vertical configuration, the 1,3‐Diox⋅⋅⋅RuC complex exhibited the largest negative *E*_ads_, followed by 1,4‐Diox⋅⋅⋅RuC, and then 1,2‐Diox⋅⋅⋅RuC. Numerically, the *E*_ads_ values of the 1,3‐Diox⋅⋅⋅, 1,4‐Diox⋅⋅⋅, and 1,2‐Diox⋅⋅⋅RuC complexes were −19.91, −19.85, and −16.27 kcal/mol, respectively, as shown in Table 1. On the other hand, the Fur adsorption over the RuC nanosheet showed the smallest negative *E*_ads_ value of −9.77 kcal/mol and an equilibrium distance of 2.45 Å (Table 1).

Compared to the vertical configuration, the Fur⋅⋅⋅RuC complex showed the highest *E*_ads_ value of −27.80 kcal/mol in the parallel configuration (Table 1). However, the 1,*n*‐Diox⋅⋅⋅RuC complexes in the parallel configuration exhibited the same order of *E*_ads_ as that in the vertical configuration. Detailedly, the negative *E*_ads_ of the 1,*n*‐Diox⋅⋅⋅RuC complexes in the parallel configuration decreased in the following order: 1,3‐Diox⋅⋅⋅ >1,4‐Diox⋅⋅⋅ >1,2‐Diox⋅⋅⋅RuC with values of −22.17, −20.88, and −20.53 kcal/mol, respectively, as shown in Table 1.

Overall, the adsorption process of the Fur and 1,*n*‐Diox molecules on the RuC nanosheet was more favorable in the parallel configuration than in the vertical one. The latter results might be explained by the interactions between the whole structure of the Fur and 1,*n*‐Diox molecules and the RuC nanosheet.

### Charge Transfer Calculation

Bader charge analysis is a useful technique to estimate the charge transfer that takes place during the adsorption process.[^55^, ^56^] Thus, the charge transfer (*Q*_t_) for the Fur/1,*n*‐Diox⋅⋅⋅RuC complexes in vertical and parallel configurations was estimated based on Equation (3) and is listed in Table 1.

As shown in Table 1, all *Q*_t_ values of the Fur/1,*n*‐Diox⋅⋅⋅RuC complexes in vertical and parallel configurations were positive, affirming the electron‐accepting nature of the examined molecules. Generally, the *Q*_t_ findings were consistent with the *E*_ads_ outcomes, at which the Fur⋅⋅⋅RuC complex in the parallel configuration showed the most significant negative *E*_ads_ and *Q*_t_ values of −27.80 kcal/mol and 0.1581 *e*, respectively (Table 1).

For the 1,*n*‐Diox⋅⋅⋅RuC complexes in both configurations, the *Q*_t_ decreased in the order of 1,4‐Diox⋅⋅⋅>1,3‐Diox⋅⋅⋅>1,2‐Diox⋅⋅⋅RuC; for instance, the *Q*_t_ values in the parallel configuration were 0.0792, 0.0776, and 0.0513 *e*, respectively (Table 1). According to Bader charge findings, the Fur/1,*n*‐Diox molecules acted as electron acceptors throughout the adsorption process over the RuC nanosheet.

**Figure 3:**
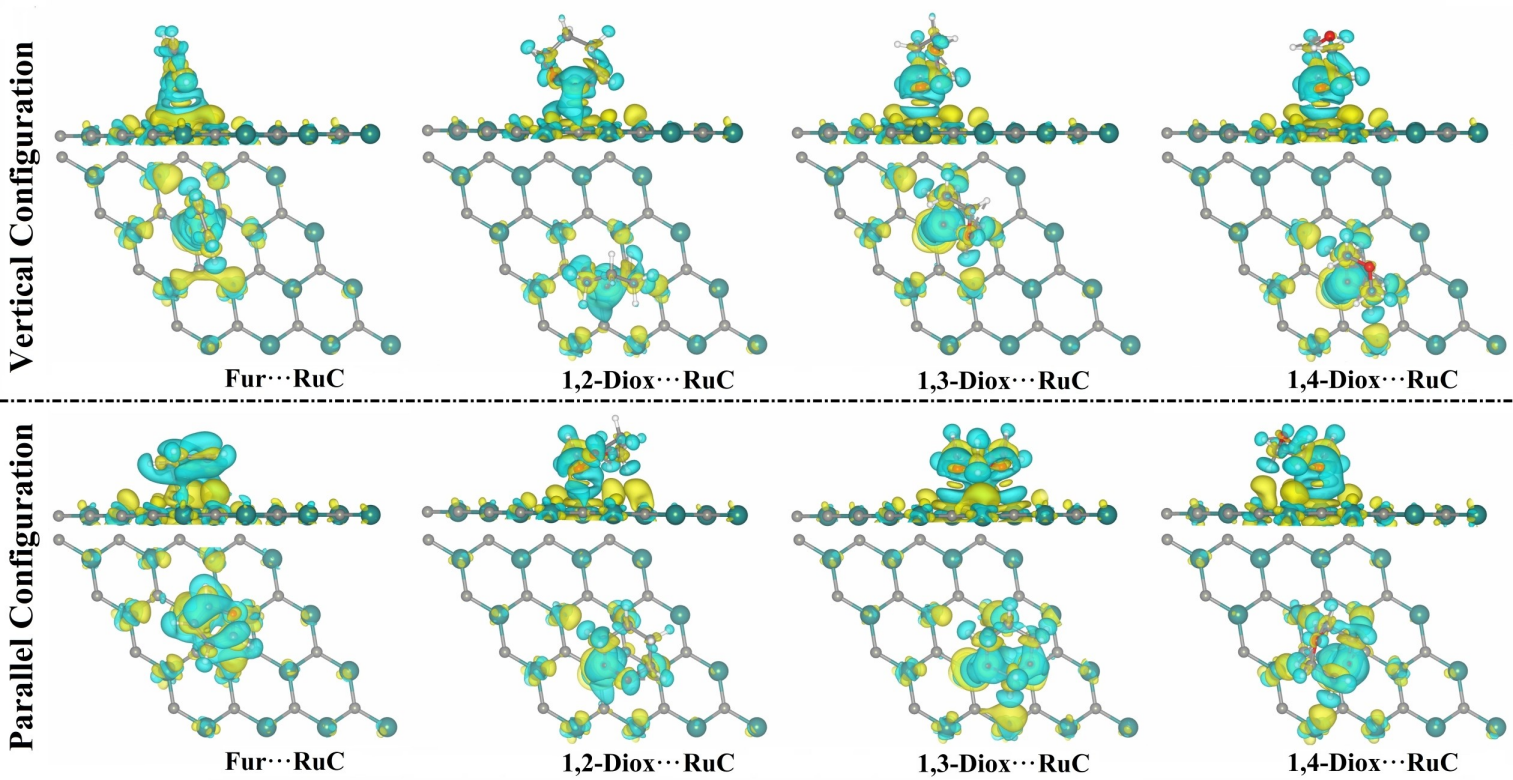
Side and top views of the charge density difference (Δ*ρ*) maps of the relaxed Fur/1,*n*‐Diox⋅⋅⋅RuC complexes (where *n*=2, 3, and 4). Yellow and cyan colors indicate electron accumulation and depletion regions, respectively.

Moreover, the charge distribution within the relaxed Fur/1,*n*‐Diox⋅⋅⋅RuC complexes in vertical and parallel configurations was plotted using charge density difference (Δ*ρ*) maps according to Equation (4) (Figure 3). As illustrated in Figure 3, positive (yellow) and negative (cyan) charges are represented by the electron accumulation and depletion regions, respectively. The Δ*ρ* maps of the Fur/1,*n*‐Diox⋅⋅⋅RuC complexes agreed with their corresponding *Q*_t_ values (Table 1). For example, the Fur⋅⋅⋅RuC complex in the parallel configuration that had the largest *Q*_t_ value of 0.1581 *e* showed the most significant amount of electron depletion and accumulation regions beneath the Fur molecule (Figure 3).

### Frontier Molecular Orbital (FMO) Analysis

**Figure 4:**
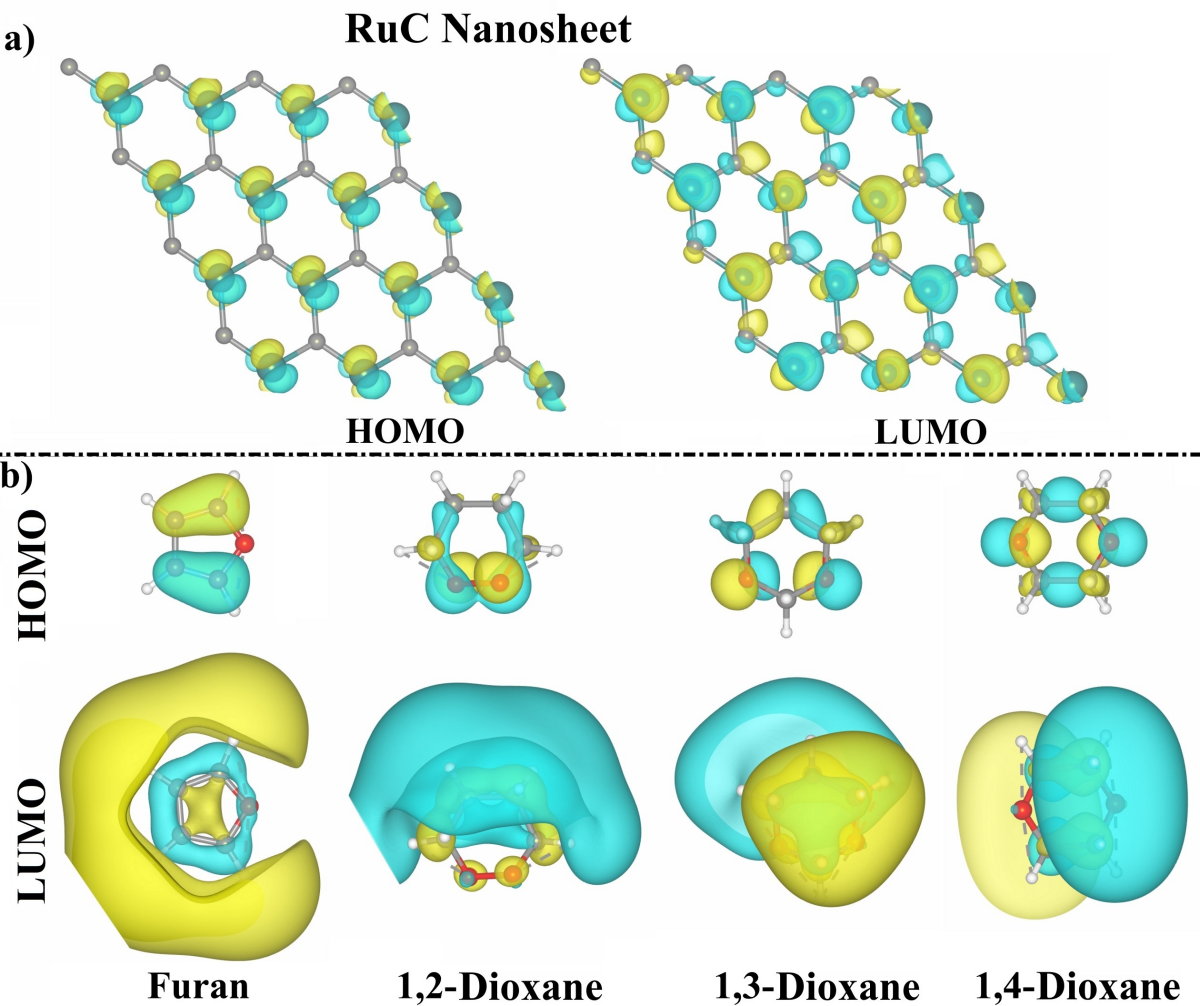
HOMO and LUMO distributions of the a) pure RuC nanosheet and b) isolated Fur/1,*n*‐Diox molecules (where *n*=2, 3, and 4).

**Figure 5:**
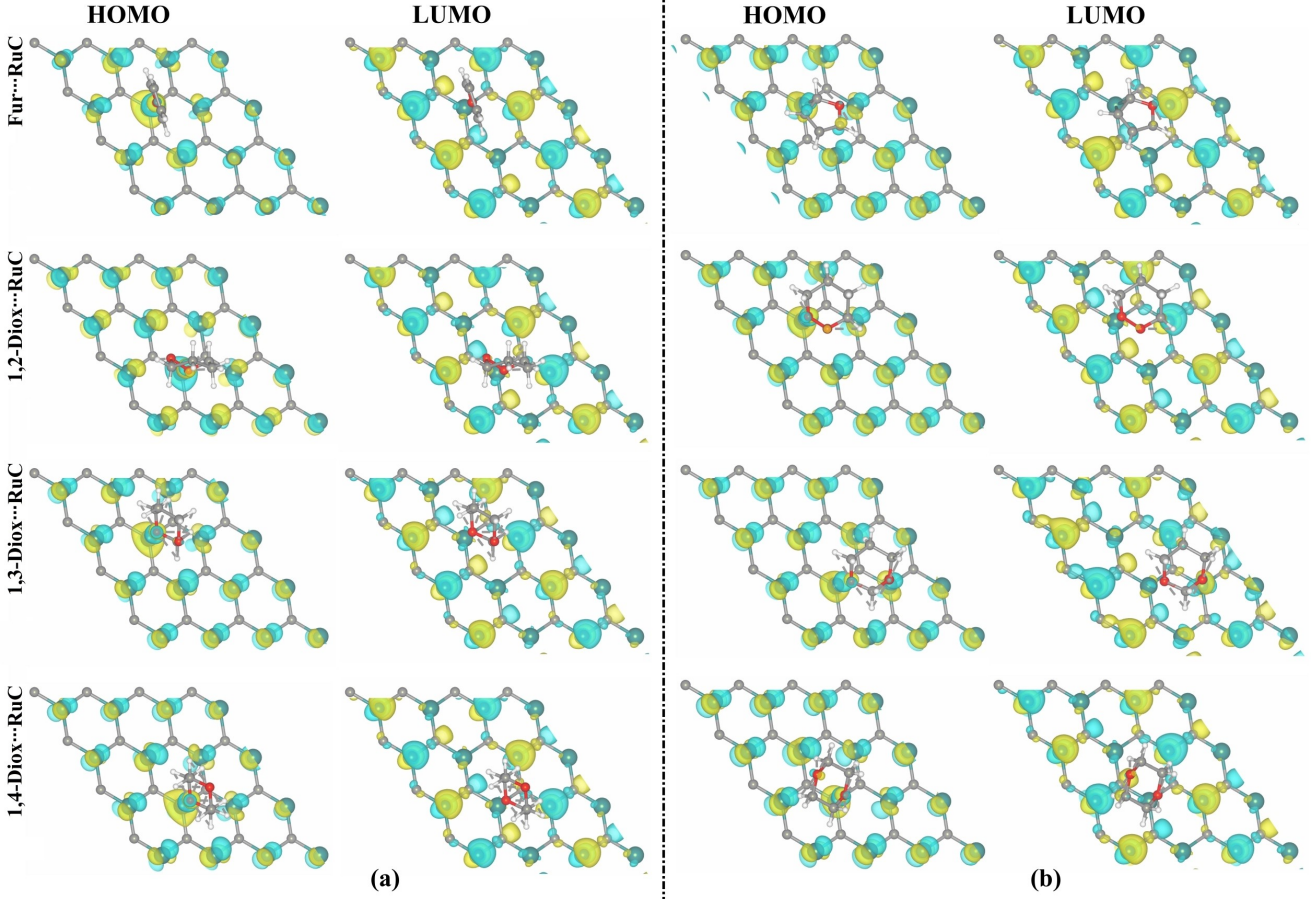
HOMO and LUMO distributions of the relaxed Fur/1,*n*‐Diox⋅⋅⋅RuC complexes (where *n*=2, 3, and 4) in (a) vertical and (b) parallel configurations.

For better comprehension of the adsorption behavior of the Fur/1,*n*‐Diox molecules over the RuC nanosheet, the FMO theory was applied. The *E*_LUMO_, *E*_HOMO_, and *E*_gap_ were calculated for the Fur/1,*n*‐Diox⋅⋅⋅RuC complexes before and following the adsorption process (Table 2). The HOMO and LUMO distributions were also produced prior to and after the adsorption process in order to assess the active electron donor and acceptor sites on the systems under investigation (Figures 4 and 5, respectively).

**Table 2:** *E*_HOMO_, *E*_LUMO_, and *E*_gap_ of the isolated adsorbent/adsorbate and the relaxed Fur/1,*n*‐Diox⋅⋅⋅RuC complexes (where *n*=2, 3, and 4) in vertical and parallel configurations.

System	*E*_HOMO_ (eV)	*E*_LUMO_ (eV)	*E*_gap_ (eV)
Isolated Systems			
Pure RuC	−3.412	−3.341	0.071
Fur	−5.540	−0.794	4.746
1,2‐Diox	−5.737	−1.008	4.729
1,3‐Diox	−5.945	−0.501	5.444
1,4‐Diox	−5.343	−0.447	4.895
Vertical Configuration			
Fur⋅⋅⋅RuC	−3.029	−3.011	0.017
1,2‐Diox⋅⋅⋅RuC	−2.704	−2.620	0.084
1,3‐Diox⋅⋅⋅RuC	−2.799	−2.716	0.082
1,4‐Diox⋅⋅⋅RuC	−2.850	−2.766	0.084
Parallel Configuration			
Fur⋅⋅⋅RuC	−2.819	−2.743	0.076
1,2‐Diox⋅⋅⋅RuC	−2.751	−2.745	0.006
1,3‐Diox⋅⋅⋅RuC	−2.699	−2.674	0.025
1,4‐Diox⋅⋅⋅RuC	−2.824	−2.812	0.012

As shown in Table 2, the *E*_HOMO_ and *E*_LUMO_ values of the isolated Fur/1,*n*‐Diox molecules, and the pure RuC nanosheet varied considerably after the adsorption process in both configurations. As an example, the *E*_HOMO_ value of the pure RuC nanosheet was −3.412 eV and changed to −2.819, −2.751, −2.699, and −2.824 eV after the adsorption of Fur, 1,2‐Diox, 1,3‐Diox, and 1,4‐Diox molecules in the parallel configuration, respectively (Table 2).

Additionally, a change in the *E*_gap_ value of the RuC nanosheet was observed after the adsorption of the Fur/1,*n*‐Diox molecules, confirming the capability of the RuC nanosheet to adsorb such molecules. For example, the *E*_gap_ value of the pure RuC nanosheet was 0.071 eV, which altered to 0.076 and 0.017 eV after adsorbing the Fur molecule in the parallel and vertical configurations, respectively (Table 2).

As seen in Figure 4, the LUMO distributions of the Fur/1,*n*‐Diox molecules were more extensive than those of the HOMO, revealing that the Fur/1,*n*‐Diox molecules acted as electron acceptors during the adsorption process on the RuC nanosheet. The latter observation was compatible with the Bader charge outcomes, where all *Q*_t_ exhibited positive values (Table 1). From Figure 5, the HOMO distributions mainly accumulated on the Ru atoms of the RuC nanosheet after adsorbing the Fur/1,*n*‐Diox molecules, demonstrating the vital role of Ru atoms in the adsorption process.

### Recovery Time

To evaluate the desorption process of Fur and 1,*n*‐Diox molecules from the RuC nanosheet, recovery time (*τ*) calculations were performed. The estimated *τ* values of the relaxed Fur/1,*n*‐Diox⋅⋅⋅RuC complexes in vertical and parallel configurations were determined (Table 3).

**Table 3:** Recovery time (*τ*, s) of the relaxed Fur/1,*n*‐Diox⋅⋅⋅RuC complexes (where *n*=2, 3, and 4) in vertical and parallel configurations at different temperatures.

Complexes	Recovery Time (s)	Complexes	Recovery Time (s)
Vertical Configuration		Parallel Configuration	
298.15 K			
Fur⋅⋅⋅RuC	1.42×10^−5^	Fur⋅⋅⋅RuC	2.25×10^8^
1,2‐Diox⋅⋅⋅RuC	8.06×10^−1^	1,2‐Diox⋅⋅⋅RuC	1.07×10^3^
1,3‐Diox⋅⋅⋅RuC	3.72×10^2^	1,3‐Diox⋅⋅⋅RuC	1.69×10^4^
1,4‐Diox⋅⋅⋅RuC	3.40×10^2^	1,4‐Diox⋅⋅⋅RuC	1.91×10^3^
310.15 K			
Fur⋅⋅⋅RuC	7.49×10^−6^	Fur⋅⋅⋅RuC	3.64×10^7^
1,2‐Diox⋅⋅⋅RuC	2.8×10^−1^	1,2‐Diox⋅⋅⋅RuC	2.79×10^2^
1,3‐Diox⋅⋅⋅RuC	1.02×10^2^	1,3‐Diox⋅⋅⋅RuC	3.98×10^3^
1,4‐Diox⋅⋅⋅RuC	9.28×10^1^	1,4‐Diox⋅⋅⋅RuC	4.92×10^2^

According to Equation 5, the *τ* increased as the negative *E*_ads_ value increased (Table 3). For instance, the Fur⋅⋅⋅RuC complex in the parallel configuration that had the largest negative *E*_ads_ of −27.80 kcal/mol showed the longest *τ* value of 2.25×10^8^ and 3.64×10^7^ s at 298.15 and 310.15 K, respectively. Based on the *τ* findings of the 1,*n*‐Diox⋅⋅⋅RuC complexes, the desorption process of the 1,3‐Diox molecule from the RuC nanosheet took the longest *τ* in the vertical and parallel configurations with values of 3.72×10^2^ and 1.69×10^4^ s at 298.15 K, respectively (Table 3). As the temperature increased to 310.15 K, the *τ* of the 1,3‐Diox⋅⋅⋅RuC complex decreased to 1.02×10^2^ and 3.98×10^3^ s in the vertical and parallel configurations, respectively.

The *τ* findings verified that the RuC nanosheet may function as a highly effective sensing device for toxic Fur/1,*n*‐Diox molecules. Obviously, the desorption process was faster at a higher temperature (i. e., 310.15 K) than at a lower temperature (i. e., 298.15 K).

### Band Structure

**Figure 6:**
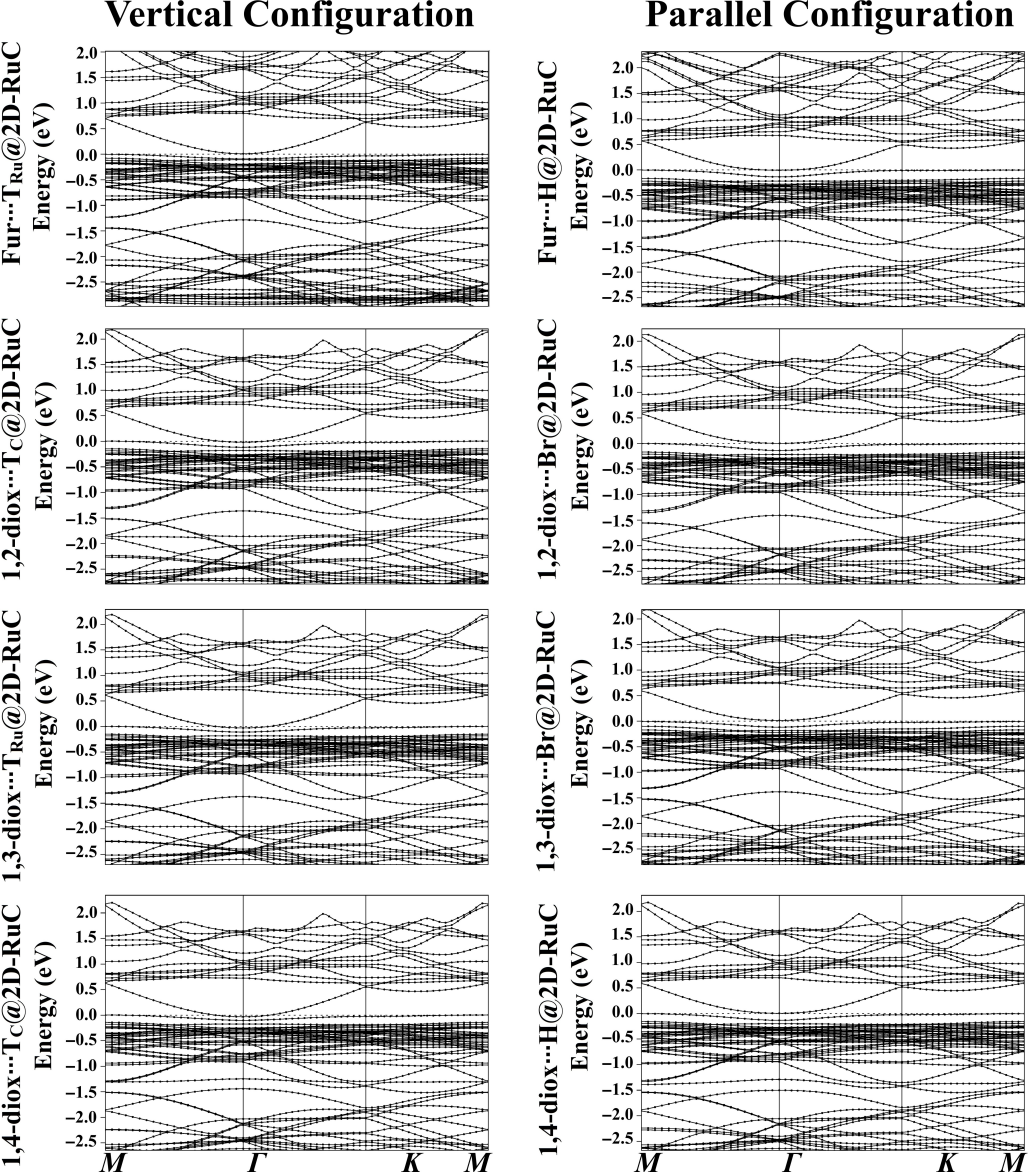
Electronic band structure along the high symmetry path of the Brillouin zone (*M‐Γ‐K‐M*) of the relaxed Fur/1,*n*‐Diox⋅⋅⋅RuC complexes (where *n*=2, 3, and 4) in vertical and parallel configurations. The dashed line at zero energy represents the Fermi level.

In order to gain a deeper understanding of the electronic features of the RuC nanosheet, band structure analysis was performed before and after the adsorption process of the Fur and 1,*n*‐Diox molecules (Figures 1 and 6, respectively).

For the pure RuC nanosheet, a narrow direct bandgap semiconductor was observed at the *Γ* point (Figure 1), which agreed with a previous study.^[28]^ After the adsorption of Fur/1,*n*‐Diox molecules over the RuC nanosheet, additional bands appeared in the band structure plot, proving that the adsorption process actually took place (Figure 6). For instance, new conduction bands were detected in the range from 1.00 to 1.65 eV, and extra valence bands were observed at −2.30 and −2.40 eV for the Fur⋅⋅⋅RuC complex in the parallel configuration (Figure 6). Moreover, valence and conduction bands shifted toward the Fermi level after the adsorption of Fur/1,*n*‐Diox molecules over the RuC nanosheet. The latter observation agreed with the *E*_gap_ findings in Table 2.

Generally, alteration in the band structures of the Fur/1,*n*‐Diox⋅⋅⋅RuC complexes was more announced in the parallel configuration compared to the vertical one, indicating that the adsorption process was more favorable in the parallel configuration.

### Density of States (DOS) Calculations

**Figure 7:**
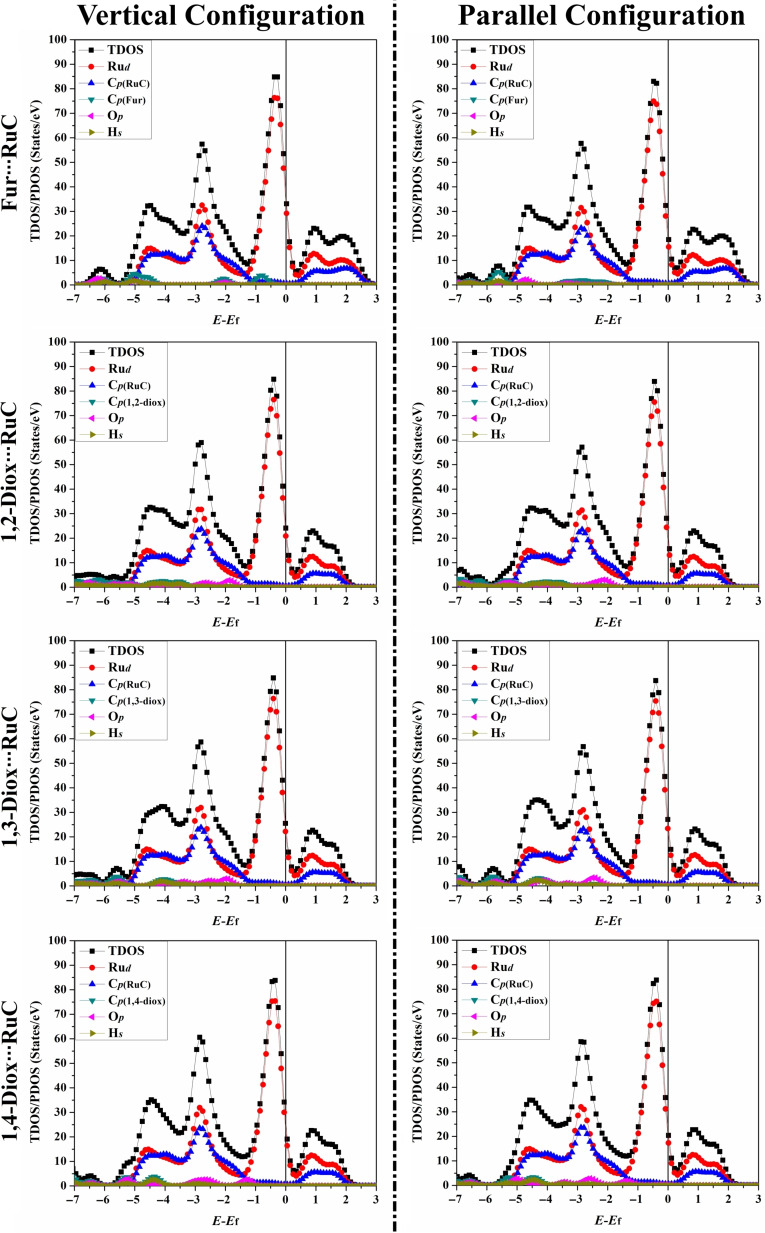
TDOS and PDOS plots of the relaxed Fur/1,*n*‐diox⋅⋅⋅RuC complexes (where *n*=2, 3, and 4). The contributions of the *d*‐, *p*‐, and *s*‐orbital of the ruthenium, carbon, oxygen, and hydrogen atoms in the adsorption process were represented by Ru_*d*_, C_*p*_, O_*p*_, and H_*s*_, respectively. The Fermi level is located at zero.

The total density of state (TDOS) and projected density of state (PDOS) were estimated to analyze the impact of the adsorption of the Fur/1,*n*‐Diox molecules on the electronic properties of the RuC nanosheet. In this spirit, TDOS and PDOS plots for the RuC nanosheet before and following the adsorption process were generated (Figures 1 and 7, respectively). According to Figure 1, the PDOS plot of the pure RuC nanosheet indicated that the *d*‐orbitals of the Ru atoms had the main source of the TDOS near the Fermi level.

As seen in Figure 7, the adsorption process originated mainly from the interaction of the Ru_*d*_ */*C_*p*_ of the RuC nanosheet and the C_*p*_/O_*p*_ of the Fur/1,*n*‐Diox molecules. For instance, in the Fur⋅⋅⋅RuC complex in the parallel configuration, new broad peaks for the C_*p*_/O_*p*_ of the Fur molecule appeared in the valence region at an energy range from −1.80 to −3.60 eV (Figure 7). Besides, in the valence region, the contributions of the H_*s*_/C_*p*_/O_*p*_ of the Fur molecule to the adsorption process were observed at an energy range from −6.00 to −7.00 eV. In this example, the C_*p*_ of the Fur molecule showed the most significant contribution to the adsorption process, supported by its overlap with the Ru_*d*_/C_*p*_ of the RuC nanosheet at the energy of −5.20 eV (Figure 7).

In the sum of TDOS/PDOS calculations, the potential for the adsorption process was confirmed by the emergence of new peaks in the Fur/1,*n*‐Diox⋅⋅⋅RuC complexes.

## Conclusions

The potential capacity of the RuC nanosheet to adsorb the Fur and 1,*n*‐Diox as highly environmental toxins was investigated employing density functional theory (DFT). The adsorption of Fur and 1,*n*‐Diox molecules on the RuC nanosheet was assessed in parallel and vertical configurations. Geometric relaxation was initially performed for the Fur/1,*n*‐Diox⋅⋅⋅RuC complexes, and then their corresponding adsorption energies (*E*_ads_) and electronic properties calculations were conducted. According to the *E*_ads_ results, Fur/1,*n*‐Diox molecules preferred to be parallelly adsorbed over the RuC nanosheet. For example, the *E*_ads_ of the Fur⋅⋅⋅RuC complexes in vertical and parallel configurations exhibited the lowest and highest negative *E*_ads_ values of −9.77 and −27.80 kcal/mol, respectively. The Bader charge results demonstrated that the RuC nanosheet served as an electron donor during the adsorption of Fur and 1,*n*‐Diox molecules, corroborated by the positive *Q*_t_ values. Based on the FMO results, the alteration of the *E*_HOMO_ and *E*_LUMO_ values of the isolated Fur/1,*n*‐Diox molecules, and the pure RuC nanosheet after the adsorption process in both configurations indicated the occurrence of the adsorption. Based on the recovery time (*τ*) calculations, the desorption of the Fur molecule from the RuC nanosheet took the longest *τ* in the parallel configuration with a value of 2.25×10^8^ s. The emergence of new peaks and bands in the TDOS/PDOS and band structure plots confirmed the ability of the RuC nanosheet to adsorb the Fur and 1,*n*‐Diox molecules. The current findings demonstrated the applicability of RuC nanosheet to be utilized as a sensor for toxic molecules, such as Fur and 1,*n*‐Diox molecules.

## Conflict of Interests

The authors declare no conflict of interest.

## Supporting information

As a service to our authors and readers, this journal provides supporting information supplied by the authors. Such materials are peer reviewed and may be re‐organized for online delivery, but are not copy‐edited or typeset. Technical support issues arising from supporting information (other than missing files) should be addressed to the authors.

Supporting Information

## Data Availability

The data that support the findings of this study are available in the supplementary material of this article.
