# Supplementary material for: Exploring the Adsorption Features of Furan and 1,n‐Dioxane as Environmental Toxins on Two‐Dimensional RuC Nanosheet: A DFT Study
Source: ChemistryOpen. 2025 Jan 29;14(6):e202400415. doi: 10.1002/open.202400415 (PMC12138048; doi:10.1002/open.202400415)
Supplement: Supplementary file 1 — Supporting Information [file OPEN-14-e202400415-s001.pdf]

ChemistryOpen

Supporting Information

Exploring the Adsorption Features of Furan and 1,*n*-Dioxane as Environmental Toxins on Two-Dimensional RuC Nanosheet: A DFT Study

Amna H. M. Mahmoud, Muhrail E. S. Aziz, Abdallah I. M. Rabee, Mohamed A. El-Tayeb, Gamal A. H. Mekhemer, Tamer Shoeib,* and Mahmoud A. A. Ibrahim*

Vertical Configuration

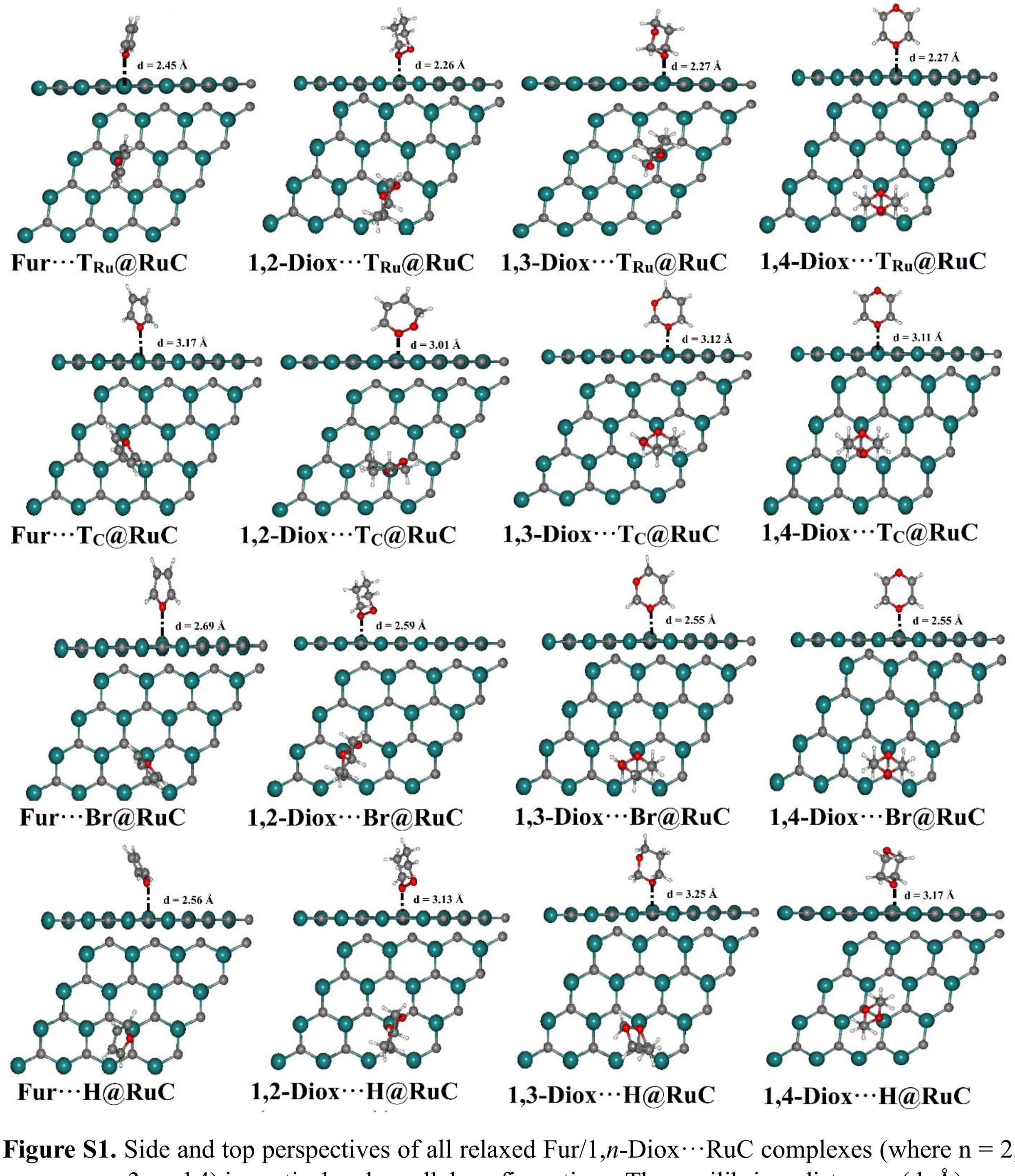

Figure S1. Side and top perspectives of all relaxed Fur/1,*n*-Diox···RuC complexes (where n = 2, 3, and 4) in vertical and parallel configurations. The equilibrium distances (d, Å) were measured between the RuC nanosheet and the oxygen atom facing its surface in the vertical configuration and between the RuC nanosheet and the center of the ring of the Fur/1,*n*-Diox molecules in the parallel configuration.

Parallel Configuration

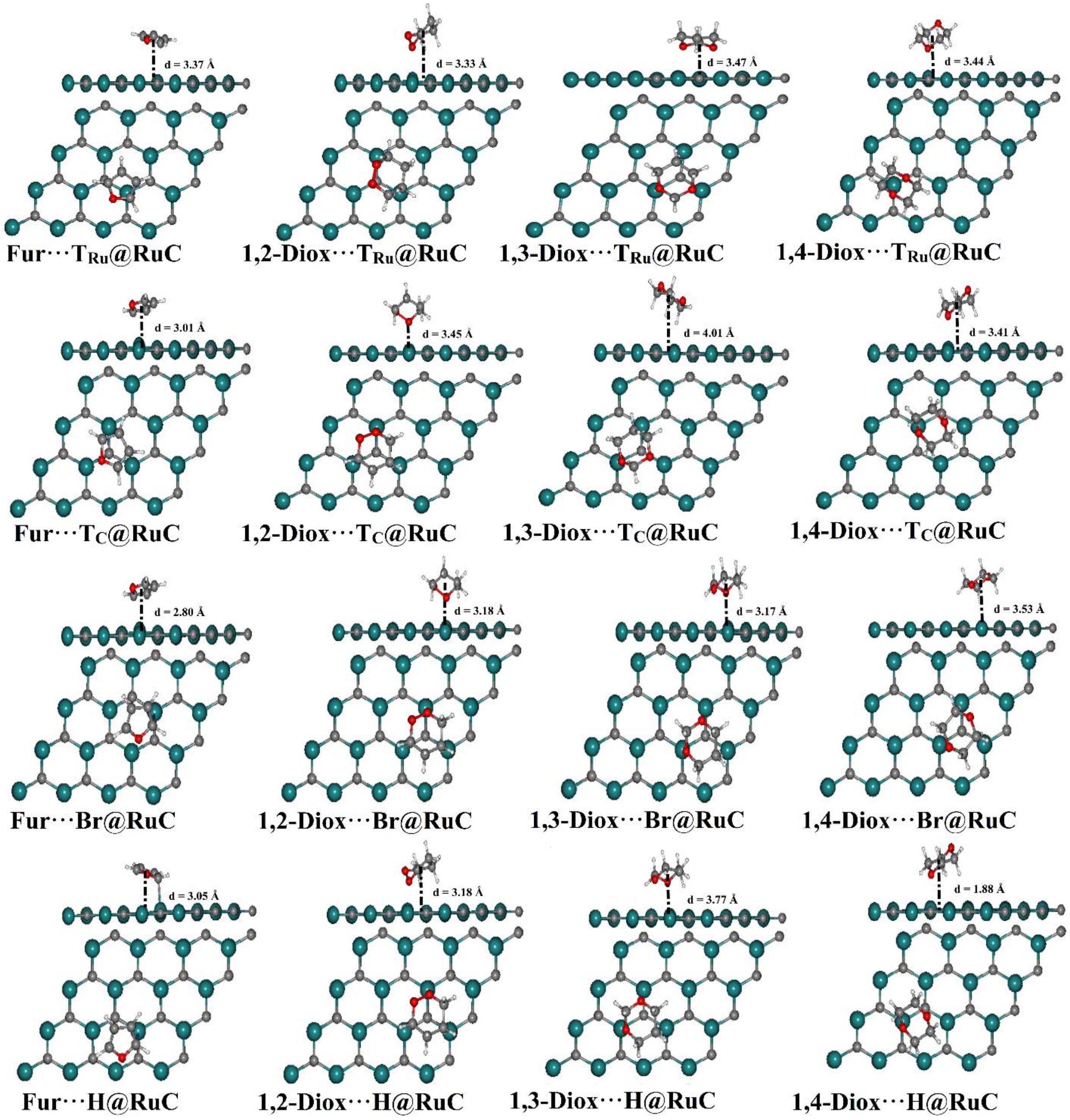

Figure S1. Continued.

Table S1. The atomic coordinates of the relaxed Fur/1,*n*-Diox···RuC complexes in vertical and parallel configurations.

**Vertical Configuration**			
Fur···RuC			
Ru	-1.7608126497	2.9305465751	0.5563031879
C	-0.1639674078	3.8529635231	0.0696650935
Ru	-3.5877388650	6.0787439356	0.5484524725
C	-1.9933761462	7.0022989166	0.0637028197
Ru	-5.4075092313	9.2278073181	0.5458178199
C	-3.8082423301	10.1509694012	0.0777541693
Ru	-7.2246361631	12.3775670290	0.5494490221
C	1.6490177071	0.6932685031	0.0647205613
Ru	1.4966667255	2.7003061614	-0.1668168460
C	3.1010968909	3.6245104684	-0.6207440646
Ru	-0.3301499455	5.8662099284	-0.1652665974
C	1.2722873634	6.7926795690	-0.6163305344
Ru	-2.1430810302	9.0153875507	-0.1528487906
C	-0.5469829334	9.9378478754	-0.6363119001
Ru	-3.9587007469	12.1605566233	-0.1523364775
C	4.9151774571	0.4773333559	-0.6376450350
Ru	4.7650038493	2.4875808861	-0.8624562362
C	6.3624158788	3.4204394848	-1.3267247040
Ru	2.9647487052	5.6572002838	-0.7705663810
C	4.5454190657	6.5712345413	-1.3192637939
Ru	1.1198335663	8.8023086033	-0.8601749820
C	2.7252384267	9.7203602017	-1.3254305793
Ru	-0.6933975857	11.9484039213	-0.8605954695
C	8.1843557279	0.2702819606	-1.3309316920
Ru	8.0329594355	2.2820099787	-1.5536383941
C	9.6314220653	3.2056168555	-2.0292041104
Ru	6.2122106178	5.4293976930	-1.5620401184
C	7.8070877032	6.3539417464	-2.0378299402
Ru	4.3895284819	8.5853893649	-1.5627180862
C	5.9880605032	9.5054587657	-2.0369368934
Ru	2.5745105567	11.7361353928	-1.5532339556
C	11.4495336427	0.0562248708	-2.0291052748
O	3.3320861532	5.8782731962	1.6445457999
C	3.1151828485	6.7044696370	3.7132011840
C	3.4643452161	5.3148510341	3.8064203563
C	3.0434736901	7.0050822604	2.3888573716
C	3.5849549786	4.8514462580	2.5335971460
H	2.9416437798	7.3938707178	4.5324920034
H	3.6093478714	4.7342397372	4.7111033811
H	2.8167970685	7.8955862031	1.8146283694
H	3.8247490586	3.8955666708	2.0840850988

1,2-Diox···RuC

Ru	0.3327427024	0.3545806600	0.2352797895
C	0.7025187115	0.5587131896	22.4420438602
Ru	-1.4960234268	3.5320055485	0.2415605753
C	-1.1397639882	3.7472216693	22.4274010716
Ru	-3.3078973476	6.6790486097	0.2536867339
C	-2.9477077983	6.8867046694	22.4194818613
Ru	-5.1214550513	9.8200433473	0.2539614951
C	-4.7600545243	10.0264200881	22.4193241488
Ru	3.6061494646	0.1481796199	-0.4472086174
C	3.9638658093	0.3688038315	21.7280423437
Ru	1.8048208642	3.3196199930	-0.3395767274
C	2.1437544084	3.5232233869	21.7419648742
Ru	-0.0447033161	6.4708049208	-0.4503471152
C	0.3239083251	6.6716204757	21.7296401743
Ru	-1.8556094845	9.6124790077	-0.4524282647
C	-1.4915246914	9.8234624276	21.7263779595
Ru	6.8721106442	-0.0529565262	-1.1457874746
C	7.2347365063	0.1560455375	21.0295523402
Ru	5.0486570728	3.0921643124	-1.1614705457
C	5.4081881259	3.3030474038	21.0163834560
Ru	3.2279518392	6.2499607598	-1.1525085071
C	3.5869725812	6.4537257119	21.0195114969
Ru	1.4123989151	9.4017140813	-1.1466610893
C	1.7746800724	9.6089924415	21.0275344317
Ru	10.1414511784	-0.2658365567	-1.8434922772
C	10.5005204233	-0.0605140785	20.3126737212
Ru	8.3116086402	2.8822161229	-1.8501858650
C	8.6691454843	3.0916323584	20.3128312286
Ru	6.4910894423	6.0284613926	-1.8559328954
C	6.8534354990	6.2371960066	20.3245019774
Ru	4.6740835965	9.1777436250	-1.8511356762
C	5.0342955374	9.3779480161	20.3088036298
O	1.0530678910	4.1500145753	2.6223792666
O	2.1160346948	3.3709371850	1.8940863921
C	2.2700827150	3.8391111188	4.7046893859
C	3.4698484622	3.3247564378	3.9006879439
C	0.9813821387	3.5730172239	3.9340397126
C	3.3922659489	3.7941040912	2.4558768402
H	2.2247590329	3.3540950642	5.6907384061
H	2.3644316649	4.9230676956	4.8727931374
H	4.4172563200	3.6730886230	4.3378163101
H	3.4903495677	2.2239249758	3.9087396609
H	0.7512680074	2.4975200881	3.8492251434
H	0.1098204347	4.0859565857	4.3703778159
H	3.4765593952	4.8855819497	2.3397165835
H	4.1344075108	3.3046723329	1.8087322857

1,3-Diox···RuC

Ru	-1.4430003214	2.9836898875	0.2584015297
C	-1.0710449641	3.1944919075	22.4770465503
Ru	-3.2738609600	6.1523003949	0.2549897358
C	-2.9086680916	6.3701035131	22.4702252806
Ru	-5.0852984120	9.3005365101	0.2634455748
C	-4.7289233162	9.5104679356	22.4229618808
Ru	-6.8994440279	12.4432170389	0.2639829272
C	0.7363386253	0.0449072382	22.4240721551
Ru	1.8276917606	2.7678960781	-0.4399436037
C	2.1846033746	2.9903475801	21.7396239048
Ru	0.0357186585	5.9487486917	-0.2939512374
C	0.3690635897	6.1436184708	21.7512219705
Ru	-1.8261992296	9.0919923079	-0.4456351253
C	-1.4538282533	9.2909129673	21.7366291433
Ru	-3.6348835430	12.2330832124	-0.4491260940
C	4.0065446316	-0.1591522625	21.7317497154
Ru	5.0925415099	2.5668129177	-1.1375541648
C	5.4532056906	2.7753265738	21.0347729008
Ru	3.2745233265	5.7146898393	-1.1485799996
C	3.6310167342	5.9232873225	21.0219356760
Ru	1.4510583488	8.8724320641	-1.1532291130
C	1.8110718240	9.0772968226	21.0207899005
Ru	-0.3660610881	12.0204741190	-1.1379159244
C	7.2716428679	-0.3749668775	21.0343724259
Ru	8.3596450685	2.3534454575	-1.8365922528
C	8.7187067789	2.5601747249	20.3268436984
Ru	6.5331258334	5.5019660467	-1.8465479311
C	6.8895243447	5.7117845581	20.3164629941
Ru	4.7141593703	8.6507678820	-1.8501095054
C	5.0757833218	8.8599251915	20.3336146979
Ru	2.8967771792	11.8010279324	-1.8468552453
C	10.5335493532	-0.5976929299	20.3177814785
O	1.6306484011	5.4895256711	3.8109323606
O	0.4286255581	6.2292006970	1.9253372404
C	0.5146183913	7.6328838447	3.9192077282
C	1.7118330175	6.8127734545	4.3806397213
C	0.4320463308	7.6104156917	2.3997642950
C	1.6172709725	5.5332992664	2.4244958254
H	-0.4082029457	7.2140259858	4.3453897374
H	0.6031964265	8.6767116038	4.2565063897
H	2.6610328744	7.2867058845	4.0656772570
H	1.7312304763	6.6727092676	5.4676599137
H	-0.4947350458	8.0589179441	2.0222794604
H	1.2861931917	8.1343912244	1.9310555178
H	2.5035642287	6.0577807699	2.0095728847
H	1.5483461378	4.5087885775	2.0445937237

1,4-Diox···RuC

Ru	-0.3311864299	0.4779922400	0.4886686588
C	1.2661656305	1.4002704091	0.0050169726
Ru	-2.1570525967	3.6261005549	0.4794803895
C	-0.5626065469	4.5508008519	-0.0038280670
Ru	-3.9763861352	6.7757404704	0.4775075690
C	-2.3775471823	7.6987499022	0.0125145901
Ru	-5.7941781856	9.9258339073	0.4793558241
C	-4.1961539519	10.8442586581	-0.0042907328
Ru	2.9273871141	0.2467550170	-0.2278171307
C	4.5352436461	1.1713472977	-0.6651586885
Ru	1.0979605223	3.4154017093	-0.2273356445
C	2.7027537542	4.3458217598	-0.6636108496
Ru	-0.7139448552	6.5639024716	-0.2179683108
C	0.8807138758	7.4879347286	-0.7041031309
Ru	-2.5284960653	9.7066927688	-0.2180909826
C	-0.9310462963	10.6259858958	-0.7039918979
Ru	6.1971110724	0.0309538294	-0.9268476905
C	7.7926073482	0.9682017913	-1.3932817495
Ru	4.4032010894	3.2113640385	-0.7909402136
C	5.9780995455	4.1205985036	-1.3836691712
Ru	2.5461380524	6.3549761042	-0.9257664244
C	4.1555439057	7.2676904411	-1.3925396929
Ru	0.7365009044	9.4965574118	-0.9280470430
C	2.3390293071	10.4215604422	-1.3968375224
Ru	9.4632708663	-0.1701825653	-1.6196875417
C	11.0612982073	0.7528564369	-2.0970766300
Ru	7.6456662156	2.9780551172	-1.6353273893
C	9.2403084197	3.9017972958	-2.1089901958
Ru	5.8224235860	6.1357828171	-1.6348905742
C	7.4193195653	7.0554226792	-2.1093415183
Ru	4.0050565976	9.2839981855	-1.6194737565
C	5.6032593890	10.2068889705	-2.0965298510
O	5.9286849483	4.0834085423	3.9811301618
O	4.8236249869	3.4494512826	1.4308970214
C	5.9954310923	2.7520176159	1.9446457408
C	5.9605044824	2.7533173537	3.4613534655
C	4.7970542548	4.8099951222	1.9523347886
C	4.7862584959	4.7712442960	3.4691264478
H	5.9476261569	1.7315254580	1.5430735616
H	6.9005761219	3.2506428938	1.5562283700
H	5.6768114030	5.3529081246	1.5650978047
H	3.8848141942	5.2742066898	1.5557148112
H	5.0786920313	2.1838366188	3.8127361366
H	6.8698315639	2.2767529657	3.8519853758
H	4.8231030118	5.7954522902	3.8645518882
H	3.8569023066	4.2846492944	3.8223150918

Parallel Configuration			
Fur···RuC			
Ru	-7.025068	12.518791	0.449873
C	1.849811	0.834813	-0.044654
Ru	-1.559962	3.066764	0.458876
C	0.030367	3.985175	-0.048158
Ru	-3.390252	6.214034	0.455600
C	-1.797156	7.140719	-0.026121
Ru	-5.213625	9.361879	0.442215
C	-3.611575	10.283700	-0.012561
Ru	-3.762109	12.289370	-0.243896
C	5.118035	0.604355	-0.706071
Ru	1.694072	2.828997	-0.289475
C	3.318498	3.751362	-0.712001
Ru	-0.140146	6.006544	-0.249104
C	1.477957	6.940615	-0.629058
Ru	-1.945742	9.151546	-0.234953
C	-0.344852	10.072889	-0.710085
Ru	-0.490615	12.084011	-0.932275
C	8.385520	0.409912	-1.401931
Ru	4.973728	2.617250	-0.921614
C	6.572672	3.558108	-1.393763
Ru	3.184629	5.811544	-0.769513
C	4.765829	6.726082	-1.404766
Ru	1.325120	8.946442	-0.906266
C	2.925388	9.862224	-1.401403
Ru	2.774476	11.874361	-1.637807
C	11.648147	0.191198	-2.122781
Ru	8.237281	2.420918	-1.629091
C	9.835130	3.342023	-2.117746
Ru	6.416310	5.570837	-1.662680
C	8.008762	6.488552	-2.144360
Ru	4.592724	8.728951	-1.655267
C	6.187087	9.652181	-2.142704
C	3.905731	4.049644	2.381384
C	5.198960	4.327683	1.979577
C	3.985170	6.079930	1.354260
H	2.077429	5.329254	2.207882
H	6.122933	3.762355	2.045383
H	3.955421	7.166435	1.396462
H	3.561736	3.125642	2.832771
C	3.136022	5.172408	2.029081
O	5.294063	5.559825	1.439363

1,2-Diox···RuC

Ru	0.2010433459	-0.1192938086	0.2063300735
C	1.7985875078	0.8005828868	-0.2789321190
Ru	-1.6147917452	3.0277564750	0.1945387801
C	-0.0050827966	3.9399891421	-0.2419606909
Ru	-3.4466338110	6.2002431477	0.1938731306
C	-1.8479069223	7.1365236624	-0.2382083506
Ru	-5.2610354406	9.3440386692	0.2071445614
C	-3.6674323275	10.2697312220	-0.2814999435
Ru	3.4669815116	-0.3277021536	-0.5073452689
C	5.0675145082	0.5990824289	-0.9804692171
Ru	1.6536699424	2.8070557498	-0.4993978433
C	3.2467930704	3.7399337402	-0.9802430703
Ru	-0.1322966848	5.9963376158	-0.3574477372
C	1.4202703728	6.8967510738	-1.0009027799
Ru	-1.9995599988	9.1391798074	-0.5042472350
C	-0.3932217464	10.0565918663	-0.9774246593
Ru	6.7357857298	-0.5362838611	-1.2030279900
C	8.3365037399	0.3862476089	-1.6756313775
Ru	4.9177537923	2.6082154467	-1.2057865412
C	6.5178221105	3.5329641516	-1.6762000024
Ru	3.0975298177	5.7533120934	-1.2188494830
C	4.6947018589	6.6792805216	-1.6844481386
Ru	1.2708444607	8.9177468971	-1.2201171654
C	2.8715516504	9.8368290428	-1.6882141338
Ru	9.9982274638	-0.7578925908	-1.9071090422
C	11.5968461032	0.1608093756	-2.3890993432
Ru	8.1875126452	2.3996719827	-1.8999924568
C	9.7832264941	3.3211434408	-2.3851240135
Ru	6.3585206969	5.5448890650	-1.9057099245
C	7.9522707250	6.4697943583	-2.3893094979
Ru	4.5348432081	8.6910527488	-1.9134128390
C	6.1357158946	9.6161436541	-2.3722808626
O	0.1360240043	6.1859091226	1.8501649892
O	1.1868908784	5.3386119339	2.5201564547
C	1.4447831823	8.1970623292	2.4101849052
C	2.5883553669	7.3144879366	2.9150526934
C	0.1175661981	7.4748378288	2.5385641309
C	2.4728946842	5.9237937367	2.3050468583
H	1.3900124844	9.1424680437	2.9689242788
H	1.6075985568	8.4651469994	1.3483419496
H	3.5626160740	7.7467826622	2.6451589183
H	2.5508150371	7.2347316522	4.0131444212
H	-0.1492983566	7.2605042327	3.5842963003
H	-0.7082296016	8.0099888368	2.0478418846
H	2.7046728060	5.9349583671	1.2148664098
H	3.1396225088	5.1842828572	2.7761439866

1,3-Diox···RuC

Ru	0.5737172979	-0.4304672242	0.2872889699
C	2.1730186284	0.4828105094	-0.1910517157
Ru	-1.2482837620	2.7338397374	0.3070556288
C	0.3593932437	3.6637437148	-0.1280326644
Ru	-3.0665838074	5.8776094243	0.3042763968
C	-1.4683245929	6.7985179789	-0.1765631252
Ru	-4.8865171833	9.0189684286	0.2918276439
C	-3.2900750855	9.9288047324	-0.2082999252
Ru	3.8276035080	-0.6675662252	-0.4517320688
C	5.4309707945	0.2842773168	-0.8555285359
Ru	2.0359891182	2.5307236240	-0.3249257852
C	3.5883012168	3.4363849573	-0.9636953478
Ru	0.1966019515	5.6715288151	-0.3981677636
C	1.7881226392	6.5945309261	-0.9003339985
Ru	-1.6203221056	8.8071817522	-0.4159251749
C	-0.0246999097	9.7411075020	-0.8917678342
Ru	7.1036020327	-0.8524122638	-1.0946823510
C	8.7010639159	0.0748379941	-1.5662801511
Ru	5.2970045219	2.3114284847	-1.0422259299
C	6.8854574082	3.2243982411	-1.5583890825
Ru	3.4490976093	5.4571188586	-1.1676942234
C	5.0624049524	6.3751402182	-1.6136925179
Ru	1.6433904291	8.6049465268	-1.1196509531
C	3.2446273630	9.5300853886	-1.5871513974
Ru	10.3732019593	-1.0626416363	-1.8013642680
C	11.9727364049	-0.1405751077	-2.2779315028
Ru	8.5504186171	2.0841853107	-1.7991429959
C	10.1474128593	3.0024431839	-2.2843111717
Ru	6.7254889983	5.2337472476	-1.8288355853
C	8.3311965957	6.1504979971	-2.2837587507
Ru	4.9110030920	8.3853306472	-1.8211304171
C	6.5099620394	9.3038190504	-2.2998964646
O	2.8835160691	2.6651878086	1.9348073465
O	5.2074844269	2.6100933160	1.5253343396
C	4.1592643586	4.7645427041	2.1050245971
C	2.9429802475	3.9766936257	2.5728864493
C	5.4317674557	3.9244710896	2.1088338436
C	4.1145611493	1.9705275864	2.1512580189
H	3.9752391709	5.1420460342	1.0762061001
H	4.2948694226	5.6493770146	2.7442790390
H	2.9633302902	3.8017735767	3.6628467611
H	2.0042040864	4.4784880200	2.3080870477
H	6.2210864242	4.3847934722	1.5013854003
H	5.8123782337	3.7692313718	3.1342870198
H	4.2917131623	1.9104114647	3.2418060250
H	4.0115677520	0.9745038041	1.7019410746

1,4-Diox···RuC

Ru	12.595804	-0.837817	-2.034928
C	1.125533	0.938217	0.275154
Ru	10.771992	2.313168	-2.038970
C	-0.695637	4.097803	0.286446
Ru	8.953173	5.462039	-2.044270
C	-2.507546	7.243633	0.296319
Ru	7.135392	8.611264	-2.041140
C	-4.328448	10.386974	0.276129
Ru	-4.486856	12.393622	0.040908
C	4.400931	0.723436	-0.372891
Ru	0.966810	2.962257	0.056889
C	2.565847	3.886503	-0.425329
Ru	-0.845027	6.110430	0.066364
C	0.749880	7.035676	-0.417429
Ru	-2.660915	9.253694	0.065151
C	-1.064868	10.177649	-0.417291
Ru	6.064460	-0.413984	-0.623227
C	7.657499	0.519217	-1.095727
Ru	4.268882	2.764238	-0.500614
C	5.849148	3.673339	-1.087999
Ru	2.416917	5.902869	-0.641499
C	4.022176	6.820577	-1.116262
Ru	0.603460	9.045068	-0.641091
C	2.203302	9.972186	-1.108385
Ru	9.328734	-0.620980	-1.329448
C	10.925695	0.301686	-1.804451
Ru	7.511270	2.529110	-1.335224
C	9.106610	3.448673	-1.817598
Ru	5.687726	5.682991	-1.352596
C	7.290111	6.602315	-1.819398
Ru	3.871120	8.831736	-1.335982
C	5.470041	9.753623	-1.808620
O	3.022795	4.310081	3.681859
O	4.691647	3.004286	1.760747
C	3.911617	2.252965	2.730576
C	2.682023	3.028777	3.156089
C	5.030537	4.312419	2.300901
C	3.787616	5.063478	2.737229
H	3.639803	1.305139	2.242625
H	4.567078	2.046962	3.593299
H	5.710861	4.146980	3.153074
H	5.563616	4.847873	1.503152
H	1.992424	3.143642	2.280215
H	2.139731	2.489209	3.943855
H	4.066096	6.004278	3.230360
H	3.163398	5.305744	1.848991

Table S2. Adsorption energies (E_{ads}, kcal/mol) and equilibrium distances (d, Å) of all the relaxed Fur/1,n-Diox···RuC complexes (where n = 2, 3, and 4) at all adsorption sites within the vertical and parallel configurations.

System	Adsorption Site	Vertical Configuration		Parallel Configuration	
		E_{ads} (kcal/mol)	d (Å)	E_{ads} (kcal/mol)	d (Å)
**Fur···RuC**	T_{Ru}	−9.77	2.45	−13.65	3.37
	T_C	−9.19	3.17	−26.09	3.01
	Br	−9.64	2.69	−27.79	2.80
	H	−9.30	2.56	−27.80	3.05
**1,2-Diox···RuC**	T_{Ru}	−16.23	2.26	−18.42	3.33
	T_C	−16.27	3.01	−20.47	3.45
	Br	−16.23	2.59	−18.36	3.33
	H	−16.25	3.13	−20.53	3.18
**1,3-Diox···RuC**	T_{Ru}	−19.91	2.27	−22.17	3.47
	T_C	−19.89	3.12	−13.31	4.01
	Br	−19.86	2.55	−22.03	3.17
	H	−18.69	2.32	−22.04	3.77
**1,4-Diox···RuC**	T_{Ru}	−19.83	2.27	−19.91	3.44
	T_C	−19.85	3.11	−20.88	3.41
	Br	−19.84	2.55	−13.81	3.80
	H	−19.85	3.17	−20.73	3.77
